# Down Syndrome in Children: A Primary Immunodeficiency with Immune Dysregulation

**DOI:** 10.3390/children11101251

**Published:** 2024-10-17

**Authors:** Aleksandra Szczawińska-Popłonyk, Natalia Popłonyk, Karina Awdi

**Affiliations:** 1 Department of Pediatric Pneumonology, Allergy and Clinical Immunology, Institute of Pediatrics, Poznań University of Medical Sciences, Szpitalna 27/33, 60-572 Poznań, Poland; 2 Student Scientific Society, Poznań University of Medical Sciences, 61-701 Poznań, Poland; 3 Student Scientific Society, English Division, Poznań University of Medical Sciences, 61-701 Poznań, Poland

**Keywords:** allergy, autoimmunity, Down syndrome, primary immunodeficiency, immune dysregulation, regulatory T cells

## Abstract

**Background:** The multisystemic features of Down syndrome (DS) in children are accompanied by immunodeficiency, making them susceptible to infections and immune dysregulation with autoimmune, allergic, inflammatory, and hematological complications. This study was aimed at a better understanding of the abnormalities within the B and T cell compartments and their correlations with clinical immunophenotypes. **Methods:** Medical records of 35 DS children were retrospectively reviewed, referring to clinical symptomatology including history of infections, immune dysregulation disorders, and humoral and cellular immune response. **Results:** While the etiology of respiratory tract infections included typical viral and bacterial pathogens, SARS-CoV2-induced inflammatory disease and syndromic immunodeficiency contributed significantly to the deterioration of the clinical course. Allergic diseases in the form of asthma, allergic rhinitis, and alimentary allergy were the most frequent manifestations of immune dysregulation and were followed by autoimmune disorders, such as Crohn’s disease, celiac disease, autoimmune thyroiditis, and alopecia, as well as inflammatory disorders, balanitis xerotica obliterans and lymphadenopathy, and a hematological disorder of myelopoiesis. Deficiency of serum immunoglobulin levels, reduced numbers of naïve B cells, and non-switched memory B cells along with low naïve T helper cells and significantly reduced regulatory T helper cells were the most prominent immune abnormalities. **Conclusions:** The loss of naïveté in B and T lymphocyte compartments with a deficiency of regulatory T cells may be underpinning pathomechanisms for the skewed immune response. The clinical immunophenotype in DS is complex and represents syndromic primary immunodeficiency with immune dysregulation.

## 1. Introduction

Trisomy 21 (OMIM 190685), aka Down syndrome (DS), is a multisystemic condition characterized by a significant clinical, epidemiological, and societal burden. With its prevalence ranging from 5.7 through 8.3 to 12.9 live births per 10,000 due to differences in geographic distributions, maternal age, prenatal testing trends, and Down syndrome-related elective terminations associated with the global tendency to reduce the overall population size [1,2,3], the syndrome remains one of the commonest genetic disorders in humans. The genetic complexity in DS is caused either by the presence of an extra copy of chromosome 21 resulting in trisomy or, in a small percentage of cases, Robertsonian translocation and attachment of the long arm of chromosome 21 to another chromosome, isochromosome or ring chromosome [4,5]. DS is characterized by a wide range of clinical phenotypic features, such as intellectual disability; craniofacial dysmorphism; congenital heart disease, most frequently atrioventricular septal defect (AVSD); congenital gastrointestinal abnormalities including Hirschsprung’s disease; duodenal atresia or imperforated anus, decreased muscle tone; increased risk of neurological complications; predominantly early development of Alzheimer dementia, and epilepsy; and endocrinopathies, such as short stature and hypothyroidism [4]. Anatomical developmental abnormalities and organ dysfunctions with airway anomalies, structural lung disease, pulmonary hypertension, obstructive sleep apnea, and aspirations are overlapping disorders and show a mutual relationship with an increased predisposition to respiratory infections in affected individuals [6,7,8]. Furthermore, innate and adaptive immune response alterations, and immune-mediated autoimmune, autoinflammatory, myelodysplastic, and lymphoproliferative disorders contribute to the syndromic immunodeficiency in DS [9,10]. It has been hypothesized that this complex phenotype is determined by a subset of dosage-sensitive genes driving alterations in diverse cellular processes. This Down Syndrome Critical Region (DSCR) is mapped to the long arm of chromosome 21 and covers 5.4 Mb containing genes regulating cellular processes, such as splicing (*RBM1* (RNA-Binding Motif 1), *U2AF1* (U2 Small Nuclear RNA Auxiliary Factor 1), *U2AF1L5* (U2 Small Nuclear RNA Auxiliary Factor 1 Like 5)), DNA methylation (*PRMT2* (Protein Arginine Methyltransferase 2), *N6AMT1* (N(6)-Adenine Specific DNA Methyltransferase)), and metabolism (*SOD1* (Superoxide Dismutase 1)). Alternatively, another hypothesis argues against the role of the critical dosage-sensitive region and suggests that multiple genetic abnormalities are responsible for the clinical phenotype in DS [11]. Complementing this view, it has been proposed that dysregulated cellular homeostasis caused by additional genetic material leads to a constitutive upregulation of genes triggering aneuploidy-associated stress and resulting in genome-wide changes in gene expression and alterations in cellular pathways. Consequently, impaired mitochondrial function, altered metabolism, epigenetic deregulation, endocytic defects, altered cell specifications, increased levels of oxidative stress, transformed proteostasis and autophagy, elevated DNA damage, and activation of the immune response play a crucial role in the pathophysiology of DS [11,12,13]. It may, therefore, be assumed that heterogeneity of deviated cellular immunoregulatory pathways on genomic, proteomic, and metabolomic levels may underpin syndromic immunodeficiency and immune dysregulation with autoimmune, inflammatory, allergic, lymphoproliferative, and myelodysplastic disorders [14,15,16]. Whereas the genetic and pathophysiological mechanisms determining the immunological defects are complex and multidirectional, it has been postulated, that overexpression of genes such as *RCAN1* (Regulator of Calcineurin 1) impairs the inhibitory effect on signal transduction mediated by the nuclear factor of activated T cells (NFAT) and has the potential to reduce the inflammatory response by stabilizing the activity of nuclear factor kappa B (NF-κB) [17].

## 2. Aim of the Study

The study was aimed at a better understanding of the abnormalities within the B and T lymph cell compartments in children with DS. We also sought to determine correlations between deficiencies in specific B and T lymphocyte subsets and the DS patients’ clinical and immunological phenotypes.

## 3. Materials and Methods

### 3.1. The Study Group

We retrospectively reviewed the medical records of a cohort of pediatric patients in whom a definitive clinical and genetic diagnosis of trisomy 21 had been established. All the children studied were referred to our Department of Pediatric Pneumonology, Allergy, and Clinical Immunology due to suspicion of a primary immunodeficiency for an in-depth evaluation of clinical phenotypes and immune parameters, from January 2022 to December 2023. The study group consisted of 35 children, 25 boys (71%) and 10 girls (29%), aged from 2 to 204 months (17 years) (mean age 45 months). A discerning retrospective analysis of medical conditions, including a history of thymectomy, upper and lower respiratory tract disorders, infectious episodes, congenital heart defects, endocrine disorders, and other malformations and organ dysfunctions that may have an impact on infectious complications has been performed in all the study participants. In all the children studied, airway aspirates were taken for RT-PCR tests for RNA or DNA of 25 respiratory viruses and also for bacterial cultures. Immunological characteristics of the participating children included immunoglobulin levels and flow cytometric analysis of the B and T cell compartments. Antibody levels and relative and absolute counts of lymphocyte subpopulations were interpreted using age-matched reference values.

### 3.2. Flow Cytometric Peripheral Blood (PB) Lymphocyte Immunophenotyping

Cells were labeled with the following murine fluorochrome-stained monoclonal antibodies: anti-CD45 FITC (fluorescein isothiocyanate), anti-CD14 PE (phycoerythrin), anti-CD19 PE, anti-CD19 PerCP (peridinin chlorophyll protein), anti-IgM FITC, anti-IgD FITC, anti-CD38 APC (allophycocyanin), anti-CD27 PE, anti-CD21 FITC, as well as anti-CD3 FITC, anti-CD4 FITC, CD45RA FITC, CD127 FITC, CD185 FITC, anti-CD8 PE, anti-CD16+CD56 PE, CD25 PE, CD31 PE, CD45RO PE, anti-CD3 PerCP, CD197 PerCP, anti-CD4 APC, and anti-CD8 APC (all Beckton-Dickinson Biosciences, Franklin Lakes, NJ, USA). The acquisition of cells and analysis were carried out with the use of the flow cytometer FACSCanto and FACSDiva software (Beckton-Dickinson, USA). With sequential gating on biparametric scattering CD45+CD14- lymphocytes, the following lymphocyte subpopulations were identified:

- CD19+ B cells, immature CD19+CD21lo, immature activated CD19+CD38loCD21lo, transitional CD19+CD38hisIgMhi, naïve CD19+CD27-sIgD+, non-switched memory CD19+CD27+sIgD+, switched memory CD19+CD27+IgD- B cells, and CD19+CD38hisIgM- plasmablasts
- CD3+ T cells, CD3+CD4+ T helper cells, CD3+CD4+CD31+CD45RA+ recent thymic emigrants, naïve CD3+CD4+CD27+CD45RA+, regulatory CD3+CD4+CD25++CD27-, central memory CD3+CD4+CD27+CD45RO+, effector memory CD3+CD4+CD27-CD45RO+, terminally differentiated CD3+CD4+CD27-CD45RA+, follicular CD3+CD4+CD185+CD45RO+, and regulatory CD3+CD4+CD45RO+CD127-CD25++ T helper cells.
- Among CD3+CD8+ cytotoxic T cells, the following subsets were distinguished: naïve CD3+CD8+CD197+CD27+CD45RA+, central memory CD3+CD8+CD197+CD27+CD45RO+, effector memory CD3+CD8+CD197-CD27-CD45RO+, and terminally differentiated CD3+CD8+CD197-CD27-CD45RA+ cells.

The relative values of PB B and T lymphocytes of the total lymphocyte population as well as B and T cell subsets were calculated. The absolute counts of all cell subsets were calculated from the PB leukocyte counts. A comparative analysis was conducted with the reference cut-off values of B and T cell subsets for pediatric populations of different age groups [18,19].

### 3.3. Statistical Analysis

To assess the significance of immune dysregulation disorders in the DS children studied, absolute counts and relative values of the analyzed lymphocyte subsets were compared between DS children and control groups using the Mann–Whitney U-test and the Student’s *t*-test in those cases when the distribution of values was normal. A significance cut-off value of *p* = 0.05 was used for each test. Differences in the distribution of incorrect values were tested using Pearson’s chi-square test and Fisher’s exact test in those cases when the assumptions for the chi-square test were not met. Again, the statistical significance level was set for each test at *p* = 0.05. All analyses were performed with the use of Statistica v. 13.3.

## 4. Results

**Clinical symptomatology.** Congenital cardiac malformations occurred in 25 DS-affected children and were the most frequent developmental abnormalities among patients in the study group. They were represented by the atrioventricular septal defect (AVSD) in 10, the atrial septal defect type II (ASD II) in 8, and the ventricular septal defect (VSD) in 6 out of 35 reported children, and ASD I, coarctation of aorta (CoA), Fallot tetralogy (ToF) and total anomalous pulmonary vein return (TAPVR), each occurring in 1 patient in the study group. As many as 13 children were thymectomized during corrective cardiac surgery.

Intestinal aganglionosis, aka Hirschsprung’s disease, was found in three of the patients, and an intrapulmonary sequestration was found in one. Both these congenital malformations and cardiovascular abnormalities contribute to increasing the risk of infections and affect developmental milestones together with immune response.

**Infections.** The predominating symptomatology indicative of immunodeficiency in the study group were recurrent episodes of respiratory tract infections, most frequently pneumonia, bronchitis, and bronchiolitis in children below the age of two years old. Airway aspirates were taken in all the children studied and the viral DNA or RNA of 9 respiratory viruses were identified by RT-PCR in as many as 17 children (49%). Most frequently, RSV and Rhinovirus-RNA were identified in airway aspirates, both in 11 children studied. Whereas in 5 out of 35 children, respiratory viruses were not found in the airway aspirates, in 2 of them, aged 14 and 17 years old (170 and 204 months, respectively), Epstein–Barr virus (EBV) DNA was found. In 12 out of 35 children, a SARS-CoV2 infection was diagnosed on RT-PCR examination, rendering this infection the most frequent viral etiology overall. All the children with COVID-19 presented with fever, severe respiratory disease, and deterioration of their cardiovascular disorders. Other coronaviruses of non-SARS serotypes, such as OC43 and HKU1, were identified in the children studied as a co-infection with other respiratory viruses.

Airway aspirates were also taken for cultures that were positive in as many as 15 children, in many of them as coinfections. The big three bacteria were *Streptococcus pneumoniae*, *Haemophilus influenzae*, and *Staphylococcus aureus*. The type and frequency of respiratory viruses and the bacterial etiology of respiratory infections in the study group are displayed in Table 1. Beyond respiratory tract infections, two children suffered from recurrent abscesses—perianal abscesses occurred in one patient and hidradenitis suppurativa in another one.

**Immune dysregulation** was observed in the study group in the form of allergic, autoimmune, inflammatory, and hematopoietic disorders. The frequency of immune dysregulation disorders and organ-specific immunopathology are summarized in Table 2.

**Figure 1:**
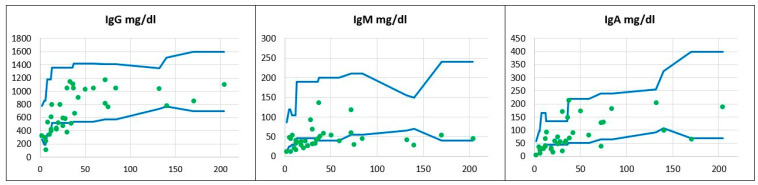
The distribution of serum immunoglobulins G, M, and A in the children studied. The immunoglobulin concentrations expressed in mg/dL in relation to the patients’ ages given in months and to age-matched reference values. Reference minimal and maximal values 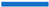, patients’ values 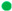. The horizontal axis shows patients’ ages (months), and the vertical axis shows patients’ results.

**Immunodiagnostics.** Immunoglobulin deficiency was the most commonly identified immunological abnormality among the reported DS patients and was observable beyond the age of 48 months when transient hypogammaglobulinemia cannot be definitely ruled out. Of note, the predominant antibody production defect was IgM deficiency, and IgM serum levels were below the age-matched reference values in as many as 23 children (66%); whereas, 12 (34%) children had normal or borderline IgM levels. IgA deficiency was the second most frequent immunoglobulin deficit, occurring in 13 out of 35 children (37%). Low IgG levels were identified in five children (14%) only and all of them were below the age of 48 months; whereas, no patients beyond the age of 4 years old had IgG hypogammaglobulinemia. The distribution of serum immunoglobulin levels related to the patients’ age is displayed in Figure 1.

**Figure 2:**
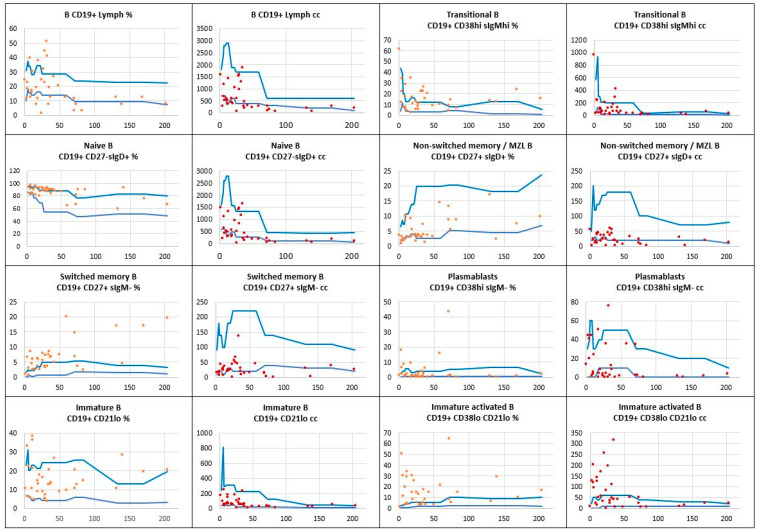
The relative counts (percentage of total lymphocytes) and absolute numbers of B cell subpopulations in a group of children with Down syndrome in relation to age-matched reference values. Reference minimal and maximal values 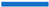, patients’ relative counts (%) 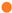, absolute numbers (cc) 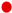. The horizontal axis shows patients’ ages (months), and the vertical axis shows patients’ results.

Within the B cell compartment, flow cytometric immunophenotyping showed a low absolute count of the total B cell pool, which occurred in 14 (40%) out of 35 children; whereas, the relative B cell count (percentage of total lymphocytes) was low in 12 (34%) of the children with Down syndrome studied. The most prominent developmental abnormalities regarding B cell subsets reported in the study group included low numbers of naïve B cells in 13 (37%) children; low memory B cell counts, among them low non-switched memory/ marginal zone B cells, in 15 (43%); and low switched memory B cells in 13 (37%) children; and finally, plasmablasts were low in 13 (37%) children. Relative and absolute counts of B cell subsets analyzed in the children studied are shown in Figure 2.

**Figure 3:**
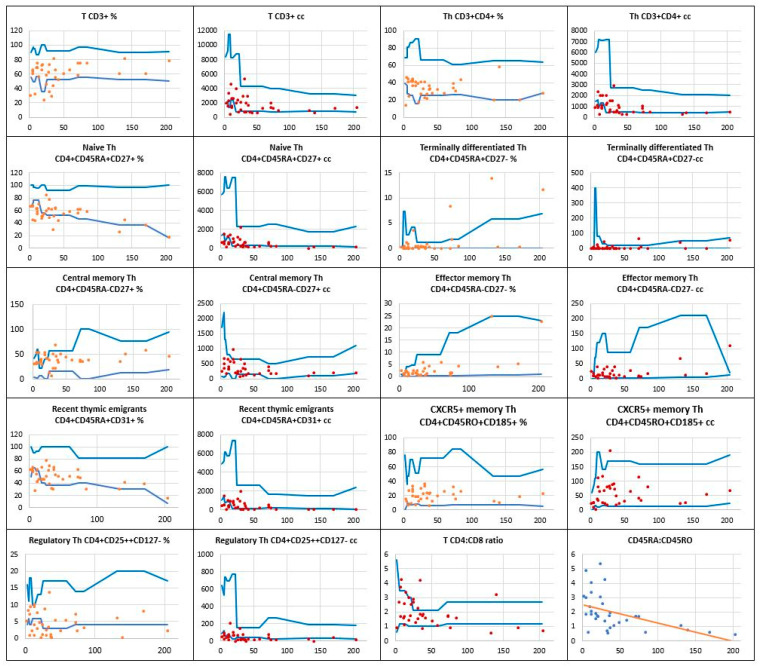
The relative counts (percentage of total lymphocytes) and absolute numbers of T CD4+ helper cell subpopulations in a group of children with Down syndrome in relation to age-matched values. The regression curve shows the naïve CD4+CD45RA+ to memory CD4+CD45RO+ T helper cell ratio. Reference minimal and maximal values 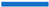, patients’ relative counts (%) 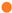, absolute numbers (cc) 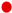. The horizontal axis shows patients’ ages (months), and the vertical axis shows patients’ results.

In Figure 3, the distribution of the total T CD3+ pool and T CD4+ helper cell subsets is displayed. Whereas the number of T CD3+ lymph cells was low in 12 children, the absolute counts of T CD4+ helper cells were below reference values in 14 children (40%). For recent thymic emigrants, both their relative counts and absolute numbers were low in 8 (23%) children. A substantial reduction in the production of naïve CD4+CD45RA+CD27+ T helper cells was seen in a significant number of the children studied, with low relative and absolute counts in 18 (51%) and 17 (49%) of them, respectively.

Meaningful defects were also assessed in the production of regulatory CD4+CD25++CD127- T helper cells. Their low relative count occurred in 18 (51%) children and the low absolute number was observable in as many as 25 (71%) patients in the study group. The regression curve displayed in Figure 3 shows the naïve CD4+CD45RA+ to memory CD4+CD45RO+ T helper cell ratio. Of note, as many as 18 children were found to have their results under the curve. In 8 children, the naïve to memory cell ratio was lower than 1, indicating a loss of T helper cell naïveté.

**Figure 4:**
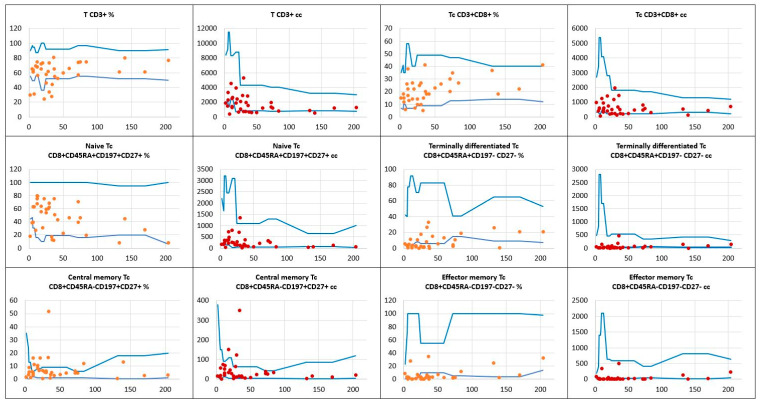
The relative counts (percentage of total lymphocytes) and absolute numbers of T CD8+ cytotoxic/suppressor cell subpopulations in a group of children with Down syndrome in relation to age-matched values. Reference minimal and maximal values 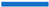, patients’ relative counts (%) 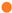, absolute numbers (cc) 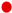. The horizontal axis shows patients’ ages (months), and the vertical axis shows patients’ results.

As opposed to T helper cell deficiencies shown in significant proportions of children with DS, abnormal distribution of the T CD8+ cytotoxic/suppressor pool was observable in a few children. The total CD8+ T cell absolute numbers were low in 3, and the relative counts in 2 children. Interestingly, relative counts of two different T CD8+ cell subsets, namely terminally differentiated and effector memory T CD8+ cells, were low in 19 (54%) and in 24 (69%) children, respectively. The results of T CD8+ cell immunophenotyping are displayed in Figure 4.

The results of peripheral blood lymphocyte B, T CD4+, and T CD8+ in all the DS children studied, in relation to age-matched reference values in healthy controls, are displayed in Supplemental Tables S1 and S2.

The statistical analysis revealed correlations of immune dysregulation, allergic, autoimmune, inflammatory, lymphoproliferative, and transient hematopoietic disorders between the study and control groups. The level of significance was *p* < 0.001 for every analyzed clinical immune dysregulation feature. Furthermore, for statistical analysis, the children studied were stratified into DS children with immune dysregulation and healthy controls to compare absolute counts and relative values of the analyzed lymphocyte subsets between groups. Again, this difference was statistically highly significant, with a *p*-value < 0.001.

## 5. Discussion

The results of our study in a pediatric group of patients with DS show a multiplicity of immunological disorders, such as severe, recurrent viral and bacterial respiratory tract infections, autoimmune manifestations, immunodeficiency with impaired antibody biosynthesis, and disturbances within B and T cell compartments. Down syndrome children present as a syndromic immunodeficiency with anatomical developmental defects and dysfunctions affecting many organs and systems, including cardiovascular, respiratory, endocrine, gastrointestinal, and neuromuscular disorders significantly overlapping with immunodeficiency, autoimmune, lymphoproliferative, and inflammatory manifestations.

Our study shows that low serum levels of IgM antibodies are the most prominent feature of humoral immunodeficiency in DS, observable in 66% of the children reported. IgM deficiency was accompanied by IgA hypogammaglobulinemia in 37% of the children studied. These results are consistent with other studies, in which low levels of IgM and IgA antibodies were demonstrated, making affected children susceptible to infections [14,20,21] and predisposing them to developing immune dysregulation disorders [17].

B cell lymphopenia was assessed in as many as 15 out of 35 children with DS. The reduced total B cell pool in patients with DS may be attributed to decreased bone marrow development and output, poor peripheral survival, and disturbed homeostasis. In contrast to other inborn errors of immunity, there is no compensatory homeostatic B cell proliferation as a response to a reduced B cell pool [22]. The results of our study showing abnormal B cell differentiation and maturation at different stages of their development are consistent with other studies [23,24]. Whereas the transitional B cell pool, which reflects the development of B cell precursors and the output from the bone marrow was not affected in our study, naïve mature B cells were significantly decreased. Since maturation of the naïve B cell pool occurs at the periphery and is an antigen and T-cell independent process, defective generation of B cells at this stage suggests an intrinsic B cell developmental defect. Referring to the low serum IgM levels in the children studied, it is worth noting that the IgM deficiency correlates with a reduced non-switched memory B cell pool, which is a germinal center-independent, predominantly IgM-producing subpopulation. Importantly, non-switched memory B cells are essential in early primary immune response, providing protection against infections caused by encapsulated bacteria and viruses, as well as in the response to vaccine antigens [25]. Although in our study switched memory B cell pool was not strikingly reduced and sustained normally in 23 out of 35 (66%) children in the study group, alterations in switched memory B cell generation were observed as a cardinal feature of impaired adaptive immunity in children with DS [22,24,26]. The development of this long-living, highly differentiated B cell subset requires T cell-dependent processes of immunoglobulin isotype switching, antigen affinity maturation, and somatic hypermutations in germinal centers. Therefore, deficiency of the switched memory B cells in DS has been suggested to be reminiscent of common variable immunodeficiency (CVID) [23,27,28,29].

In our study, significant developmental disorders of the T CD4+ helper cells were observable. These alterations included a reduction of the total T CD4+ helper cell pool in 40% of the children studied and low naïve CD4+CD45RA+CD27+ T helper cell absolute and relative counts in 46% and 51% of DS children studied. In as many as 23% of the children participating in our study, the CD45RA: CD45RO ratio was lower than 1, reflecting the loss of naïveté and decline in the size of newly generated naïve T CD4+ helper cells, which may implicate their functional consequences of immunosenescence observable in DS [30,31]. It has been proposed that the induction of cellular senescence may contribute to early thymic involution and immune dysregulation. This hypothesis has been supported by the analysis of senescence and cellular damage markers in thymocytes and peripheral T cells, such as increased beta-galactosidase activity, increased levels of the cell cycle inhibitor p16, telomere length, and increased levels of reactive oxygen species reflecting increased oxidative stress [31]. The further considered pathophysiological mechanism of T cell dysfunction in DS is T cell exhaustion, a state of altered immunological profile induced by chronic inflammation due to chronic infection or malignancy related to inflammatory cytokine signaling cascades [32]. In DS, the immune phenotype with increased expression of the programmed death receptor PD-1, the immune checkpoint protein CD160, and the immunoregulatory receptor SLAM (signaling lymphocyte activation molecule) or CD244 T cell inhibitory molecules have been associated with T cell exhaustion [32,33]. Noteworthy, the evidence for T cell exhaustion has also been found in other inborn errors of immunity (IEI) characterized by impaired T cell functions, such as proliferation and cytokine expression, and increased susceptibility to apoptosis, in which genetic underpinnings predispose to immune dysregulation, such as variants in *LRBA* (Lipopolysaccharide (LPS) Responsive Beige-Like Anchor Protein), *CTLA-4* (Cytotoxic T Cell-Associated Protein 4), *PI3KR1* (Phosphoinositide-3-Kinase Regulatory Subunit 1), and *PIK3CD* (Phosphatidylinosito-4,5-Biphosphate 3-Kinase Catalytic Subunit Delta) [34]. Furthermore, in our study, we demonstrated novel alterations of the immune response in DS children, a reduced CD4+CD25++CD127- regulatory T cell (Treg) subset which is an important factor modulating the cellular activity and intensity of the immune response. Although Tregs are thought to be abundant in DS individuals, effector T CD4+ and T CD8+ cells seem to be resistant to Treg-mediated suppression resulting in overproduction of cytokines amplifying autoimmune and autoinflammatory response [35].

It may, therefore, be assumed, that the above-mentioned alterations within T and B cell compartments, including features of lymphocyte senescence and exhaustion, contribute significantly to an increased susceptibility to infections, and predisposition to autoimmune, autoinflammatory, and malignant disorders associated with DS patients in our study group. Another aspect of symptomatology related to immune dysregulation in DS is an aberrant inflammatory cytokine profile with elevated serum levels of acute phase markers, such as interleukin (IL)-1β, IL-6, tumor necrosis factor (TNF)-α, and interferon (IFN)-γ [36,37,38]. The IFN hyperactivity has been hypothesized to be a background for an autoimmunity-prone state, phenotypically presented in our patients with autoimmune thyroiditis, alopecia, celiac, and Crohn’s disease [39,40,41]. Interferonopathy in Down syndrome children was also reported to be associated with the development of acute immune dysregulation, life-threatening clinical condition, hemophagocytic lymphohistiocytosis treated with favorable outcomes targeting interferon pathway with emapalumab [42].

An insight into the pathophysiology of immune dysregulation in DS raises important questions about uncovering its genetic and epigenetic underpinnings. Interestingly, several genes involved in the regulation of the immune response and associated with the biosynthesis of IFN receptor subunits, are localized on chromosome 21. Type III IFN and cytokines IL-10, IL-22, and IL-26 are ligands for this receptor, thereby linking the genetic overdosage in trisomy 21 with autoimmunity and autoinflammation in DS [35]. Autoimmune regulator, the *AIRE* gene, is also encoded on the 21 chromosome and is overrepresented in trisomy 21, yet its altered thymic expression in DS seems to dysregulate the negative selection of autoreactive thymocytes. Consequently, less accurate controlling of the thymic process may predispose DS children to autoimmune disorders [35,43]. The studies on the immunogenetic background of DS were also focused on the epigenetic characteristics of DS. Significant alterations in the DNA methylation profile were observed in DS patients that can be functionally linked to various immunophenotypic aspects. DNA methylation signature in blood cells was associated, among others, with the *RUNX1* (Runt-related Transcription Factor 1) gene involved in the development of hematopoietic cells and thus, important in myeloid leukemogenesis, or the *EBF4* (Early B Cell Factor 4) gene playing a pivotal role in B cell maturation. Altered DNA methylation was demonstrated in genes downstream of the PI3K/Akt/mTOR signaling pathway regulating the cell cycle and the activity of multiple cellular processes, in particular, promoting cellular growth and survival [44]. Epigenetic mechanisms affecting gene expression are associated with micro RNA (miRNA) deregulation in DS. Overexpression of miR-99a, let-7c, miR-125b-2, miR-155, and miR-802 shown in the brain and heart of DS individuals, are involved in the regulation of Treg development and macrophage responses [35].

Referring to the numerousness and multiplicity of respiratory tract viral infections in the children studied, the results of viral studies clearly pointed to a high frequency of respiratory viruses as etiological factors of infectious airway episodes in DS children. The spectrum of etiologies included the big three of respiratory viruses, with respiratory syncytial virus (RSV), rhinovirus, and, surprisingly, bocavirus. Of note, the most frequent infection among children studied was COVID-19 and it had the most devastating effect on respiratory and cardiovascular conditions, with systemic inflammatory response syndrome (SIRS) [45,46,47]. Children with Down syndrome are particularly vulnerable to a severe course of COVID-19 due to syndromic immunodeficiency associated with immune dysregulation [48], yet in immunocompromised children, the susceptibility to pneumonia due to other coronaviruses, such as OC43 and HKU1, is also increased [49,50].

In adult DS patients, an epidemiological study [51] has demonstrated a low risk of viral infections, while in children with DS, respiratory viruses are the leading etiological factors of airway infections, contributing to a high rate of hospitalizations, respiratory failure, and fatalities, as well as having a strong impact on neurodevelopmental scores [52]. Beyond defects in immunity, meaningful contributory factors make DS children vulnerable to frequent and severe infectious respiratory episodes, such as neuromuscular disorders, psychomotor delay, gastroesophageal reflux, upper airway abnormalities with macroglossia and dysfunctional swallowing, structural lung disease, and congenital heart disease with altered pulmonary blood flow [6,7,8,52,53]. While increased expression of the genes for alpha/beta or type I interferon type I (IFN-I) receptors, *IFNAR1* and *IFNAR2*, and enhanced signaling of type I interferon pathways might be assumed to serve as protective factors due to their crucial role in controlling viral infections. Defective regulation of IFN-I mechanisms may be an additional explanation for the impaired response to respiratory viruses in children with DS [54,55].

The T helper 2 (Th2)-skewed immune response with increased levels of IL-4 and IL-13, cytokines that regulate various aspects of allergic inflammation [56] was observed in a proportion of participants in our study. This may be another pathogenetic factor contributing to the susceptibility to infections in children with DS.

It is also worth noting that viral infections have also been proposed as inducing and modulating factors of autoimmune diseases. Molecular mimicry, viral antigen epitope spreading, bystander activation, and immortalization of B cells have been hypothesized as mechanisms of the viral-induced immunopathology of loss of tolerance and the development of autoimmune diseases. Influenza A virus; herpes viruses, such as Epstein–Barr virus, herpes simplex viruses, and human herpes virus 6; as well as Coxsackie viruses [57] and SARS-CoV-2, which were revealed with a high frequency in the DS children studied, may play a contributory role in the triggering and sustaining autoimmunity [58].

Predisposition to abnormal hematopoiesis in children with DS is perceived as another manifestation of the immune dysregulation phenotype, resulting from mutual interactions between epigenetic, oncogenic, chromosomal, and cellular homeostasis mechanisms [59]. Children with DS have a significantly increased risk of both myeloid and lymphoblastic leukemia compared to the general pediatric population [60,61]. A unique neonatal myeloproliferative disorder, observable in as many as 4 patients in our study group, transient abnormal myelopoiesis (TAM), manifests with circulating blasts having morphological and immunological features of the megakaryocytic lineage, posing a risk of developing myeloid leukemia associated with Down syndrome (ML-DS) [62,63]. The preleukemic, myelodysplastic phase of TAM in blasts bearing trisomy 21 is driven by variants in the key hematopoietic transcription factor *GATA1* gene. While in the majority of infants with DS, TAM resolves spontaneously, 5 to 10% of them are found to have variants in cohesin, protein CCCTC-binding factor *CTCF* gene, oncogenes belonging to the *RAS* family, and genes of the Janus Kinase and Signal Transducer and Activator of Transcription *JAK*/*STAT4* pathway that induce progressing to ML-DS [60,61,62,63]. Consequently, in DS, an integrated multidisciplinary approach and care as well as the development of further comprehensive research strategies with special emphasis on immunodeficiency and immune dysregulation are required [64,65].

## 6. Conclusions

In the last decades, significant progress has been made in the field of immunodiagnostic cell phenotyping and immunogenetics contributing to a better understanding of the immunodeficiency and immune dysregulation phenomena in children with DS. Their immunophenotypes show multiple and complex lymphoproliferative and myeloproliferative alterations due to chromosomal aneuploidy, single gene variants, epigenetic dysregulation, and molecular dysfunctions. Consequently, immunodeficiency with infectious complications and immune dysregulation in the form of autoimmune, allergic, inflammatory, and hematopoietic disorders are observable in pediatric DS patients. IgM hypogammaglobulinemia with B and T cell lymphopenia and reduced numbers of naïve B and T helper cells in children with DS along with low numbers of cytotoxic effector memory T cells are crucial immunological abnormalities predisposing to infectious diseases. In this study, we have also demonstrated that in addition to the loss of naïveté of T helper cells, low numbers of regulatory T cells may contribute to immune dysregulation disorders in children with DS. Non-immunological phenotyping features, being integral parts of DS and having a significant impact on immune deficiency and immune dysregulation disorders, place this multisystemic condition among syndromic primary immunodeficiency diseases.

## Figures and Tables

**Table 1:** Infectious etiologies of respiratory tract diseases and their frequencies in DS children.

Infectious Etiology			
Type of Infection		Frequency	
**Viral**	SARS-CoV2	N = 12 (34%)	Total number of infected children N = 17 (49%)
	RSV	N = 11 (31%)	
	Rhinovirus	N = 11 (31%)	
	Bocavirus	N = 6 (17%)	
	Adenovirus	N = 3 (9%)	
	Coronavirus OC43	N = 3 (9%)	
	Influenza virus A/B	N = 2 (6%)	
	Parainfluenza 3 and 4	N = 2 (6%)	
	Coronavirus HKU1	N = 1 (3%)	
**Bacterial**	Streptococcus pneumoniae	N = 12 (34%)	Total number of infected children N = 15 (43%)
	Haemophilus influenzae	N = 9 (26%)	
	Staphylococcus aureus	N = 8 (23%)	
	Escherichia coli	N = 6 (18%)	
	Klebsiella pneumoniae	N = 4 (11%)	
	Pseudomonas aeruginosa	N = 1 (3%)	

**Table 2:** Frequency of immune dysregulation disorders and organ-specific immunopathology in DS children.

Immune Dysregulation Disorders			
Type of Disorder		Frequency	
**Allergic**	Asthma	N = 8 (23%)	Total N = 17 (49%)
	Food allergy	N = 8 (23%)	
	Allergic rhinitis	N = 1 (3%)	
**Autoimmune**	Thyroiditis	N = 1 (3%)	Total N = 4 (11%)
	Alopecia	N = 1 (3%)	
	Celiac disease	N = 1 (3%)	
	Crohn’s disease	N = 1 (3%)	
**Inflammatory**	Balanitis xerotica obliterans	N = 2 (6%)	Total N = 6 (18%)
	Hidradenitis suppurativa	N = 2 (6%)	
	Lymphadenopathy	N = 2 (6%)	
**Hematopoietic**	Transient abnormal myelopoiesis	N = 4 (11%)	Total N = 4 (11%)

## Data Availability

The datasets generated during and/or analyzed during the current study are available from the corresponding author upon reasonable request due to privacy.

## Supplementary Materials

The following supporting information can be downloaded at: https://www.mdpi.com/article/10.3390/children11101251/s1, Table S1: Absolute numbers (cc) and relative counts, i.e., percentage of total lymphocytes (%) of the B cell subsets in all the DS children studied; Table S2: Absolute numbers (cc) and relative counts, i.e., percentage of total lymphocytes (%) of the T CD4+ and CD8+ cell subsets in all the DS children studied.

## Abbreviations

AIRE	Autoimmune Regulator
Akt	Protein kinase B (PKB)
APC	Allophycocyanin
AVSD	Atrioventricular septal defect
CoA	Coarctation of aorta
CVID	Common variable immunodeficiency
CTCF	CCCT-Binding Factor
CTLA-4	Cytotoxic T Cell-Associated Protein 4
DS	Down Syndrome
DSCR	Down Syndrome Critical Region
EBF4	Early B Cell Factor 4
EBV	Epstein-Barr virus
FITC	Fluorescein isothiocyanate
GATA1	Globin Transcription Factor 1
IEI	Inborn errors of immunity
IFN	Interferon
IFNAR	Interferon alpha/beta receptor
JAK	Janus Kinase
LRBA	Lipopolysaccharide Responsive Beige-Like Anchor Protein
ML-DS	Myeloid leukemia associated with Down syndrome
mTOR	Mammalian Target of Rapamycin
NFAT	Nuclear factor of activated T cells
NF-κB	Nuclear factor kappa B
N6AMT1	N(6)-Adenine Specific DNA Methyltransferase
PD-1	Programmed death receptor
PE	Phycoerythrin
PerCP	Peridinin chlorophyll protein
PI3K	Phosphoinositide-3 Kinase
PIK3CD	Phosphatidylinositol-4,5-Biphosphate 3-Kinase Catalytic Subunit Delta
PIK3R1	Phosphoinositide-3-Kinase Regulatory Subunit 1
PRMT2	Protein Arginine Methyltransferase 2
RBM1	RNA-Binding Motif 1 (human spermatogenesis candidate gene)
RCAN1	Regulator of Calcineurin 1
RUNX1	Runt-related Transcription Factor 1
SLAM	Signaling lymphocyte activation molecule
SOD1	Superoxide Dismutase 1
STAT4	Signal Transducer and Activator of Transcription 4
TAM	Transient abnormal myelopoiesis
TAPVR	Total anomalous pulmonary vein return
TNF	Tumor necrosis factor
ToF	Tetralogy of Fallot
U2AF1	U2 Small Nuclear RNA Auxiliary Factor 1
U2AF1L5	U2 Small Nuclear RNA Auxiliary Factor 1 Like 5
VSD	Ventricular septal defect
