# Supplementary material for: Down Syndrome in Children: A Primary Immunodeficiency with Immune Dysregulation
Source: Children (Basel). 2024 Oct 17;11(10):1251. doi: 10.3390/children11101251 (PMC11506678; doi:10.3390/children11101251)
Supplement: Supplementary file 1 [file children-11-01251-s001.zip › children-3225960-supplementary.pdf]

Supplemental Table S1. Absolute numbers (cc) and relative counts, ie. percentage of total lymphocytes (%) of the B cell subsets in all the DS children studied

No.	Sex	Age	WBC	LYMPH	B CD19+ Lymph						Transitional B CD19+ CD38hi sIgMhi					
		m	cc	%	%	N. min	N. max	cc	N. min	N. max	%	N. min	N. max	cc	N. min	N. max
1	f	2	12500	49	24	10,0	30,7	1567	700	1800	60,9	4,1	43,9	954	50	570
2	f	5	7980	27	11	18,4	37,5	253	700	2400	11,2	11,4	38,4	28	130	940
3	m	6	8420	60	22	15,7	34,1	1137	700	2800	21,5	7,2	19,7	244	100	300
4	m	6	12000	31	18	15,7	34,1	670	700	2800	33,7	7,2	19,7	226	100	300
5	m	8	10990	11	39	15,7	34,1	505	700	2800	7,0	7,2	19,7	35	100	300
6	m	10	6530	69	12	13,9	28,2	565	400	2900	17,2	3,6	12,7	97	20	210
7	m	12	4740	39	17	13,9	28,2	324	400	2900	27,8	3,6	12,7	90	20	210
8	m	12	8560	24	17	13,9	28,2	366	400	2900	6,7	3,6	12,7	25	20	210
9	m	12	12620	26	15	13,9	28,2	500	400	2900	7,1	3,6	12,7	35	20	210
10	m	13	10050	44	30	13,9	28,2	1396	400	2900	3,8	3,6	12,7	53	20	210
11	f	18	6320	45	19	16,1	34,4	556	600	1900	34,3	3,3	16,5	191	30	200
12	m	18	10600	45	7	16,1	34,4	385	600	1900	1,8	3,3	16,5	7	30	200
13	m	20	8510	38	11	16,1	34,4	360	600	1900	15,5	3,3	16,5	56	30	200
14	m	22	17910	12	37	16,1	34,4	1014	600	1900	10,5	3,3	16,5	106	30	200
15	f	25	8860	39	1	14,1	28,5	18	400	1700	11,8	3,1	12,3	2	20	200
16	m	25	14130	16	24	14,1	28,5	558	400	1700	4,5	3,1	12,3	25	20	200
17	m	28	15500	29	32	14,1	28,5	1517	400	1700	7,9	3,1	12,3	120	20	200
18	f	30	10040	65	22	14,1	28,5	1547	400	1700	10,6	3,1	12,3	164	20	200
19	m	30	9820	20	44	14,1	28,5	936	400	1700	5,3	3,1	12,3	50	20	200
20	f	33	15500	15	51	14,1	28,5	1275	400	1700	21,0	3,1	12,3	267	20	200
21	f	34	10010	45	41	14,1	28,5	1847	400	1700	22,5	3,1	12,3	416	20	200
22	m	36	5770	60	7	14,1	28,5	247	400	1700	21,5	3,1	12,3	53	20	200
23	f	37	5000	46	19	14,1	28,5	456	400	1700	26,2	3,1	12,3	119	20	200
24	m	38	7030	16	12	14,1	28,5	145	400	1700	15,2	3,1	12,3	22	20	200
25	f	42	3540	30	26	14,1	28,5	293	400	1700	19,6	3,1	12,3	57	20	200
26	m	50	4040	25	20	14,1	28,5	204	400	1700	8,4	3,1	12,3	17	20	200
27	m	59	13000	13	12	14,1	28,5	229	400	1700	9,9	3,1	12,3	23	20	200
28	m	72	5600	40	11	9,7	23,7	255	300	600	4,1	4,6	8,3	10	10	40
29	f	72	5360	47	3	9,7	23,7	80	300	600	9,0	4,6	8,3	7	10	40
30	m	75	4130	37	7	9,7	23,7	115	300	600	14,3	4,6	8,3	16	10	40
31	m	84	4360	23	3	9,7	23,7	30	300	600	5,7	4,6	8,3	2	10	40
32	m	132	4840	29	12	9,4	22,8	169	200	600	13,6	1,4	13,0	23	10	60
33	m	140	2830	22	7	9,4	22,8	48	200	600	12,7	1,4	13,0	6	10	60
34	m	170	6010	30	12	9,4	22,8	232	200	600	23,2	1,4	13,0	54	10	60
35	m	204	6140	27	8	7,2	22,5	138	100	600	14,8	0,9	5,7	20	0	30

No.	Naive B CD19+ CD27-sIgD+						Non-switched memory / MZL B CD19+ CD27+ sIgD+						Switched memory B CD19+ CD27+ sIgM-					
	%	N. min	N. max	cc	N. min	N. max	%	N. min	N. max	cc	N. min	N. max	%	N. min	N. max	cc	N. min	N. max
1	93,4	85,7	94,8	1463	580	1600	3,5	1,7	6,5	55	20	50	1,1	0,3	1,5	17	10	90
2	82,8	82,1	95,2	209	620	2120	0,7	2,5	8,7	2	20	200	6,5	0,1	2,5	16	10	179
3	97,2	85,5	93,4	1105	600	2590	1,8	2,8	7,4	20	30	120	0,6	0,5	2,1	7	20	140
4	89,6	85,5	93,4	600	600	2590	3,1	2,8	7,4	21	30	120	2,9	0,5	2,1	19	20	140
5	82,1	85,5	93,4	414	600	2590	1,5	2,8	7,4	8	30	120	8,5	0,5	2,1	43	20	140
6	89,2	76,5	94,7	504	360	2800	2,5	3,0	10,7	14	20	140	4,6	0,3	2,1	26	10	100
7	94,1	76,5	94,7	305	360	2800	2,1	3,0	10,7	7	20	140	2,4	0,3	2,1	8	10	100
8	80,8	76,5	94,7	296	360	2800	10,1	3,0	10,7	37	20	140	6,0	0,3	2,1	22	10	100
9	92,3	76,5	94,7	461	360	2800	8,4	3,0	10,7	42	20	140	6,0	0,3	2,1	30	10	100
10	94,6	76,5	94,7	1321	360	2800	3,3	3,0	10,7	46	20	140	2,5	0,3	2,1	35	10	100
11	92,6	68,1	89,3	515	490	1560	2,8	4,1	13,9	16	30	170	3,6	0,7	3,4	20	30	180
12	78,8	68,1	89,3	303	490	1560	9,1	4,1	13,9	35	30	170	6,1	0,7	3,4	23	30	180
13	86,3	68,1	89,3	311	490	1560	5,6	4,1	13,9	20	30	170	6,0	0,7	3,4	22	30	180
14	92,7	68,1	89,3	940	490	1560	3,4	4,1	13,9	34	30	170	2,6	0,7	3,4	26	30	180
15	90,2	54,0	88,4	16	280	1330	1,6	2,7	19,8	1	20	180	5,4	0,8	5,0	1	20	220
16	86,7	54,0	88,4	483	280	1330	3,8	2,7	19,8	21	20	180	7,7	0,8	5,0	43	20	220
17	90,9	54,0	88,4	1379	280	1330	3,0	2,7	19,8	46	20	180	3,6	0,8	5,0	55	20	220
18	92,1	54,0	88,4	1425	280	1330	3,7	2,7	19,8	57	20	180	3,1	0,8	5,0	48	20	220
19	85,2	54,0	88,4	798	280	1330	3,6	2,7	19,8	34	20	180	7,1	0,8	5,0	66	20	220
20	89,3	54,0	88,4	1139	280	1330	3,2	2,7	19,8	41	20	180	3,6	0,8	5,0	46	20	220
21	89,2	54,0	88,4	1647	280	1330	2,9	2,7	19,8	54	20	180	7,5	0,8	5,0	139	20	220
22	82,0	54,0	88,4	202	280	1330	7,2	2,7	19,8	18	20	180	8,5	0,8	5,0	21	20	220
23	88,4	54,0	88,4	403	280	1330	3,9	2,7	19,8	18	20	180	6,6	0,8	5,0	30	20	220
24	88,6	54,0	88,4	129	280	1330	1,3	2,7	19,8	2	20	180	7,0	0,8	5,0	10	20	220
25	87,1	54,0	88,4	255	280	1330	3,6	2,7	19,8	11	20	180	4,9	0,8	5,0	14	20	220
26	85,7	54,0	88,4	175	280	1330	3,1	2,7	19,8	6	20	180	7,5	0,8	5,0	15	20	220
27	63,6	54,0	88,4	145	280	1330	14,2	2,7	19,8	32	20	180	20,1	0,8	5,0	46	20	220
28	80,9	47,3	77,0	207	130	460	8,7	5,2	20,4	22	20	100	6,7	1,7	5,4	17	40	140
29	65,2	47,3	77,0	52	130	460	13,0	5,2	20,4	10	20	100	14,8	1,7	5,4	12	40	140
30	89,4	47,3	77,0	103	130	460	5,5	5,2	20,4	6	20	100	3,5	1,7	5,4	4	40	140
31	89,1	47,3	77,0	27	130	460	8,7	5,2	20,4	3	20	100	2,2	1,7	5,4	1	40	140
32	59,6	51,3	82,5	100	120	430	16,9	4,6	18,2	28	20	70	16,9	1,5	4,0	28	30	110
33	92,0	51,3	82,5	44	120	430	2,3	4,6	18,2	1	20	70	4,6	1,5	4,0	2	30	110
34	74,6	51,3	82,5	173	120	430	7,4	4,6	18,2	17	20	70	17,2	1,5	4,0	40	30	110
35	64,7	48,4	79,7	89	60	470	9,7	7,0	23,8	13	10	80	19,8	1,1	3,3	27	20	90

No.	Plasmablasts CD19+ CD38hi sIgM-						Immature B CD19+ CD21lo						Immature activated B CD19+ CD38lo CD21lo					
	%	N. min	N. max	cc	N. min	N. max	%	N. min	N. max	cc	N. min	N. max	%	N. min	N. max	cc	N. min	N. max
1	0,9	0,2	2,7	14	0	30	10,2	6,7	23,1	160	40	240	0,7	0,4	2,2	11	0	20
2	17,6	0,4	3,3	44	0	40	32,8	7,1	31,1	83	50	810	50,4	0,5	2,9	127	0	50
3	1,8	0,2	4,0	20	0	60	21,5	6,2	20,3	244	70	290	10,7	0,4	3,3	122	0	50
4	6,1	0,2	4,0	41	0	60	5,7	6,2	20,3	38	70	290	30,0	0,4	3,3	201	0	50
5	8,7	0,2	4,0	44	0	60	5,4	6,2	20,3	27	70	290	19,5	0,4	3,3	98	0	50
6	0,2	0,4	5,5	1	0	30	10,1	4,3	23,1	57	40	310	4,0	0,5	4,5	23	0	40
7	1,4	0,4	5,5	5	0	30	20,8	4,3	23,1	67	40	310	4,8	0,5	4,5	16	0	40
8	0,7	0,4	5,5	3	0	30	38,0	4,3	23,1	139	40	310	34,0	0,5	4,5	125	0	40
9	0,7	0,4	5,5	3	0	30	36	4,3	23,1	180	40	310	29,1	0,5	4,5	145	0	40
10	1,7	0,4	5,5	24	0	30	4,1	4,3	23,1	57	40	310	3,5	0,5	4,5	49	0	40
11	8,9	0,5	3,0	50	10	40	8,7	5,1	21,3	48	50	310	30,4	1,0	5,7	169	10	60
12	0,0	0,5	3,0	0	10	40	14,5	5,1	21,3	56	50	310	14,5	1,0	5,7	56	10	60
13	1,2	0,5	3,0	4	10	40	12,7	5,1	21,3	46	50	310	8,6	1,0	5,7	31	10	60
14	0,8	0,5	3,0	8	10	40	17,7	5,1	21,3	179	50	310	25,4	1,0	5,7	258	10	60
15	1,1	0,6	4,0	1	10	50	8,4	4,1	24,4	1	20	230	5,6	1,7	5,4	1	10	60
16	0,5	0,6	4,0	3	10	50	16,1	4,1	24,4	90	20	230	14,8	1,7	5,4	83	10	60
17	0,0	0,6	4,0	0	10	50	4,8	4,1	24,4	73	20	230	12,9	1,7	5,4	196	10	60
18	2,3	0,6	4,0	36	10	50	7,0	4,1	24,4	108	20	230	2,4	1,7	5,4	37	10	60
19	0,4	0,6	4,0	4	10	50	5,1	4,1	24,4	48	20	230	2,7	1,7	5,4	25	10	60
20	0,0	0,6	4,0	0	10	50	3,5	4,1	24,4	45	20	230	3,5	1,7	5,4	45	10	60
21	4,1	0,6	4,0	76	10	50	12,1	4,1	24,4	223	20	230	5,9	1,7	5,4	109	10	60
22	0,8	0,6	4,0	2	10	50	20,1	4,1	24,4	50	20	230	17,1	1,7	5,4	42	10	60
23	2,6	0,6	4,0	12	10	50	12,6	4,1	24,4	57	20	230	8,4	1,7	5,4	319	10	60
24	5,7	0,6	4,0	8	10	50	13,3	4,1	24,4	19	20	230	3,8	1,7	5,4	6	10	60
25	0,0	0,6	4,0	0	10	50	26,6	4,1	24,4	7	20	230	14,7	1,7	5,4	43	10	60
26	0,6	0,6	4,0	1	10	50	8,4	4,1	24,4	17	20	230	3,6	1,7	5,4	7	10	60
27	15,7	0,6	4,0	36	10	50	9,1	4,1	24,4	21	20	230	21,5	1,7	5,4	49	10	60
28	0,6	0,6	5,3	2	0	30	10,2	5,9	25,8	26	20	120	8,6	2,3	10,0	22	10	40
29	43,0	0,6	5,3	35	0	30	20,0	5,9	25,8	16	20	120	64,0	2,3	10,0	51	10	40
30	1,4	0,6	5,3	2	0	30	12,4	5,9	25,8	14	20	120	5,2	2,3	10,0	6	10	40
31	0,0	0,6	5,3	0	0	30	14,3	5,9	25,8	4	20	120	14,3	2,3	10,0	4	10	40
32	0,3	0,6	6,5	1	0	20	10,1	2,9	13,2	17	10	50	6,8	2,7	8,7	11	10	30
33	0,0	0,6	6,5	0	0	20	27,8	2,9	13,2	13	10	50	29,4	2,7	8,7	14	10	30
34	0,5	0,6	6,5	1	0	20	18,8	2,9	13,2	44	10	50	10,1	2,7	8,7	23	10	30
35	2,5	0,4	2,4	3	0	10	20,1	3,2	19,6	28	10	40	16,4	1,6	10,0	23	10	20

Supplemental Table S2. Absolute numbers (cc) and relative counts, ie. percentage of total lymphocytes (%) of the T CD4+ and CD8+ cell subsets in all the DS children studied

No.	Sex	Age	WBC	LYMPH	T CD3+ Lymph						Th CD3+CD4+					
		m	cc	%	%	N. min	N. max	cc	N. min	N. max	%	N. min	N. max	cc	N. min	N. max
1	f	2	12500	49	30	55	90	1918	1900	8400	13	39	69	845	1500	6000
2	f	5	7980	27	65	49	97	1506	2200	9200	46	37	69	1070	1600	6500
3	m	6	8420	60	64	49	95	3321	1400	11500	44	27	81	2284	1000	7200
4	m	6	12000	31	61	49	95	2269	1400	11500	41	27	81	930	1000	7200
5	m	8	10990	11	31	49	95	394	1400	11500	24	27	81	305	1000	7200
6	m	10	6530	69	69	56	87	4519	2400	8300	42	25	86	1977	1300	7100
7	m	12	4740	39	68	56	87	1310	2400	8300	44	25	86	848	1300	7100
8	m	12	8560	24	68	56	87	1423	2400	8300	37	25	86	770	1300	7100
9	m	12	12620	26	75	56	87	2512	2400	8300	42	25	86	1055	1300	7100
10	m	13	10050	44	56	56	87	2607	2400	8300	43	25	86	2002	1300	7100
11	f	18	8320	45	65	36	100	1920	700	8800	41	16	91	1222	400	7200
12	m	18	10600	45	72	36	100	3897	700	8800	37	16	91	2021	400	7200
13	m	20	8510	38	73	36	100	2423	700	8800	39	16	91	1313	400	7200
14	m	22	17910	12	24	36	100	644	700	8800	17	16	91	455	400	7200
15	f	25	8860	39	58	52	92	2084	850	4300	41	25	66	1459	500	2700
16	m	25	14130	16	46	52	92	1085	850	4300	27	25	66	635	500	2700
17	m	28	15500	29	62	52	92	2903	850	4300	33	25	66	1536	500	2700
18	f	30	10040	65	73	52	92	5274	850	4300	41	25	66	2914	500	2700
19	m	30	9820	20	34	52	92	721	850	4300	22	25	66	478	500	2700
20	f	33	15500	15	28	52	92	700	850	4300	21	25	66	525	500	2700
21	f	34	10010	45	44	52	92	1982	850	4300	27	25	66	535	500	2700
22	m	36	5770	60	81	52	92	2845	850	4300	32	25	66	1132	500	2700
23	f	37	5000	46	65	52	92	1563	850	4300	36	25	66	876	500	2700
24	m	38	7030	16	55	52	92	655	850	4300	33	25	66	399	500	2700
25	f	42	3540	30	52	52	92	573	850	4300	28	25	66	316	500	2700
26	m	50	4040	25	60	52	92	616	850	4300	31	25	66	316	500	2700
27	m	59	13000	13	66	52	92	1218	850	4300	28	25	66	520	500	2700
28	m	72	5600	40	57	55	97	1319	770	4000	34	26	61	776	400	2500
29	f	72	5360	47	74	55	97	1978	770	4000	39	26	61	1033	400	2500
30	m	75	4130	37	75	55	97	1210	770	4000	30	26	61	485	400	2500
31	m	84	4360	23	75	55	97	824	770	4000	43	26	61	478	400	2500
32	m	132	4840	29	61	52	90	883	850	3200	20	20	65	285	400	2100
33	m	140	2830	22	80	52	90	553	850	3200	58	20	65	403	400	2100
34	m	170	6010	30	61	52	90	1175	850	3200	20	20	65	375	400	2100
35	m	204	6140	27	77	50	91	1306	780	3000	28	28	64	481	500	2000

No.	Naive Th CD4+ CD45RA +						Memory Th CD4+ CD45RO+		CD45 RA:RO	Naive Th CD4+RA+CD27+					
	%	N. min	N. max	cc	N. min	N. max	%	cc		%	N. min	N. max	cc	N. min	N. max
1	75,7	66	100	640	1300	5700	24,3	205	3,12	66,7	66	100	563	1300	5700
2	75,0	73	100	803	1600	6000	25,0	268	3,00	66,6	73	100	713	1600	6000
3	82,8	77	97	1891	800	7600	17,1	390	4,84	68,9	77	97	1574	800	7600
4	64,0	77	97	595	800	7600	36,0	335	1,78	46,9	77	97	436	800	7600
5	38,0	77	97	116,0	800	7600	62,0	189	0,61	44,00	77	97	144	800	7600
6	70,0	77	96	1384	1100	6400	29,9	591	2,34	64,3	77	96	1270	1100	6400
7	77,0	77	96	653	1100	6400	23,0	195	3,35	63,0	77	96	535	1100	6400
8	67,0	77	96	516	1100	6400	33,0	254	2,03	58,7	77	96	452	1100	6400
9	64,1	77	96	676	1100	6400	35,9	378	1,78	55,3	77	96	583	1100	6400
10	80,0	77	96	1601	1100	6400	20,0	400	4,00	70,5	77	96	1411	1100	6400
11	65,0	56	100	794	200	7500	35,0	428	1,86	47,1	56	100	575	200	7500
12	59,6	56	100	1205	200	7500	40,4	817	1,48	50,6	56	100	1023	200	7500
13	62,9	56	100	826	200	7500	37,1	487	1,7	51,6	56	100	677	200	7500
14	62,4	56	100	284	200	7500	37,6	171	1,66	55,6	56	100	253	200	7500
15	84,2	52	92	1229	300	2300	15,8	231	5,33	84,3	52	92	1230	300	2300
16	75,3	52	92	478	300	2300	24,7	157	3,05	60,4	52	92	383	300	2300
17	72,0	52	92	1106	300	2300	28,0	430	2,57	63,6	52	92	976	300	2300
18	81,0	52	92	2360	300	2300	19,00	554	4,26	77,6	52	92	2262	300	2300
19	57,5	52	92	275	300	2300	42,5	203	1,35	49,1	52	92	234	300	2300
20	44,0	52	92	231	300	2300	66,0	346	0,66	62,1	52	92	321	300	2300
21	33,0	52	92	177	300	2300	67,0	359	0,49	30,0	52	92	161	300	2300
22	63,9	52	92	723	300	2300	36,1	409	1,77	50,8	52	92	575	300	2300
23	66,3	52	92	581	300	2300	33,7	295	1,97	63,1	52	92	553	300	2300
24	48,0	52	92	192	300	2300	52,0	208	0,92	65,9	52	92	275	300	2300
25	51,5	52	92	163	300	2300	48,5	153	1,06	44,3	52	92	140	300	2300
26	55,6	52	92	176	300	2300	44,4	140	1,25	54,2	52	92	172	300	2300
27	59,0	52	92	307	300	2300	41,0	213	1,44	57,7	52	92	300	300	2300
28	62,8	46	99	487	200	2500	37,2	289	1,69	55,8	46	99	433	200	2500
29	62,0	46	99	641	200	2500	38,0	393	1,63	61,8	46	99	638	200	2500
30	62,3	46	99	302	200	2500	37,7	183	1,65	62,8	46	99	305	200	2500
31	38,0	46	99	182	200	2500	62,0	297	0,61	59,0	46	99	282	200	2500
32	51,1	37	97	146	200	1700	48,9	139	1,04	26,5	37	97	75	200	1700
33	43,6	37	97	176	200	1700	56,4	227	0,77	46,6	37	97	188	200	1700
34	37,9	37	97	142	200	1700	62,1	233	0,61	37,7	37	97	141	200	1700
35	31,8	16	100	153	100	2300	68,2	328	0,47	18,8	16	100	90	100	2300

No.	Terminally differentiated Th CD4+RA+CD27-						Central memory Th CD4+RA-CD27+						Effector memory Th CD4+RA-CD27-					
	%	N. min	N. max	cc	N. min	N. max	%	N. min	N. max	cc	N. min	N. max	%	N. min	N. max	cc	N. min	N. max
1	0,2	0,0	0,1	1	0	1	30,5	4	41	258	90	1700	2,7	0,0	1,0	23	0	26
2	0,0	0,0	0,3	0	0	5	32,5	2	51	348	53	2200	0,9	0,1	0,6	10	2	21
3	0,4	0,0	7,3	9	0	400	29,9	2	59	684	83	1300	0,7	0,1	1,0	17	1	72
4	0,0	0,0	7,3	0	0	400	52,3	2	59	486	83	1300	1,2	0,1	1,0	11	1	72
5	0,0	0,0	7,3	0	0	400	53,4	2	59	163	83	1300	2,6	0,1	1,0	8	1	72
6	1,2	0,0	2,7	23	0	80	32,6	7	22	644	190	800	2,0	0,1	4,0	40	0	120
7	1,0	0,0	2,7	8	0	80	35,6	7	22	302	190	800	0,4	0,1	4,0	3	0	120
8	0,2	0,0	2,7	1	0	80	39,5	7	22	304	190	800	1,6	0,1	4,0	12	0	120
9	0,0	0,0	2,7	0	0	80	36,5	7	22	385	190	800	1,4	0,1	4,0	15	0	120
10	0,0	0,0	2,7	0	0	80	28,7	7	22	574	190	800	0,7	0,1	4,0	14	0	120
11	0,0	0,0	4,1	0	0	30	51,7	0	40	631	1	650	1,2	0,0	4,7	15	1	150
12	0,0	0,0	4,1	0	0	30	48,3	0	40	977	1	650	1,0	0,0	4,7	21	1	150
13	3,6	0,0	4,1	47	0	30	42,4	0	40	556	1	650	2,5	0,0	4,7	33	1	150
14	0,4	0,0	4,1	2	0	30	42,5	0	40	193	1	650	1,5	0,0	4,7	7	1	150
15	0,5	0,0	1,2	7	0	20	12,5	15	56	182	160	660	2,7	0,3	9,0	40	3	89
16	0,3	0,0	1,2	2	0	20	38,0	15	56	241	160	660	1,3	0,3	9,0	8	3	89
17	0,0	0,0	1,2	0	0	20	5,5	15	56	65	160	660	0,5	0,3	9,0	6	3	89
18	0,2	0,0	1,2	4	0	20	21,7	15	56	632	160	660	0,5	0,3	9,0	16	3	89
19	0,5	0,0	1,2	2	0	20	49,8	15	56	238	160	660	0,7	0,3	9,0	3	3	89
20	0,3	0,0	1,2	13	0	20	21,6	15	56	113	160	660	5,5	0,3	9,0	28	3	89
21	0,3	0,0	1,2	2	0	20	67,5	15	56	361	160	660	2,2	0,3	9,0	12	3	89
22	0,2	0,0	1,2	2	0	20	45,7	15	56	517	160	660	3,3	0,3	9,0	38	3	89
23	0,0	0,0	1,2	0	0	20	36,4	15	56	319	160	660	0,5	0,3	9,0	4	3	89
24	1,0	0,0	1,2	4	0	20	32,5	15	56	129	160	660	1,1	0,3	9,0	4	3	89
25	0,2	0,0	1,2	0	0	20	51,3	15	56	162	160	660	4,2	0,3	9,0	13	3	89
26	0,0	0,0	1,2	0	0	20	43,9	15	56	139	160	660	1,9	0,3	9,0	6	3	89
27	0,1	0,0	1,2	1	0	20	36,6	15	56	190	160	660	5,5	0,3	9,0	29	3	89
28	8,3	0,0	1,8	64	0	20	34,6	0	100	268	4	510	1,4	0,3	18,0	11	3	170
29	0,0	0,0	1,8	0	0	20	37,7	0	100	389	4	510	0,5	0,3	18,0	6	3	170
30	1,7	0,0	1,8	8	0	20	33,9	0	100	164	4	510	1,7	0,3	18,0	8	3	170
31	0,4	0,0	1,8	2	0	20	36,6	0	100	175	4	510	4,1	0,3	18,0	19	3	170
32	14,0	0,0	5,8	40	0	50	34,8	13	76	99	120	740	24,7	0,5	25,0	70	5	210
33	0,2	0,0	5,8	1	0	50	49,5	13	76	200	120	740	3,8	0,5	25,0	15	5	210
34	0,2	0,0	5,8	1	0	50	57,2	13	76	214	120	740	5,0	0,5	25,0	19	5	210
35	11,7	0,0	6,8	56	0	70	47,0	18	95	226	180	1100	22,5	1,0	23,0	108	13	22

No.	Recent thymic emigrants CD4+CD45RA+CD31+						CXCR5+ memory Th CD4+CD45RO+CD185+						Regulatory Th CD4+CD25++CD127-					
	%	N. min	N. max	cc	N. min	N. max	%	N. min	N. max	cc	N. min	N. max	%	N. min	N. max	cc	N. min	N. max
1	64,3	50	100	544	1000	4900	15,3	0,4	76	25	1	60	5	4	16	42	83	640
2	60,3	64	94	645	1400	5200	13,0	7	35	29	6	89	8,3	6	11	89	120	530
3	67,3	65	90	1536	800	6200	7,4	9	47	12	15	110	0,8	4	18	19	60	740
4	61,4	65	90	571	800	6200	8,2	9	47	27	15	110	6,9	4	18	64	60	740
5	29,8	65	90	91	800	6200	28,6	9	47	5	15	110	9,5	4	18	29	60	740
6	56,7	61	93	1120	900	5800	8,9	7	70	38	11	200	2,2	6	10	43	63	690
7	64,0	61	93	543	900	5800	20,9	7	70	34	11	200	0,9	6	10	8	63	690
8	44,4	61	93	342	900	5800	34,0	7	70	83	11	200	9,4	6	10	72	63	690
9	43,4	61	93	458	900	5800	30,0	7	70	113	11	200	4,1	6	10	43	63	690
10	46,5	61	93	931	900	5800	25,6	7	70	67	11	200	10,1	6	10	202	63	690
11	66,4	40	100	811	170	7400	7,6	8	51	29	15	140	7,7	6	13	94	20	770
12	46,8	40	100	947	170	7400	18,3	8	51	119	15	140	2,8	6	13	58	20	770
13	47,1	40	100	618	170	7400	18,3	8	51	68	15	140	4,7	6	13	62	20	770
14	47,5	40	100	216	170	7400	11,9	8	51	24	15	140	0,0	6	13	0	20	770
15	78,4	37	100	1145	190	2600	30,7	6	72	80	13	170	4,2	3	17	62	39	150
16	51,9	37	100	330	190	2600	15,5	6	72	21	13	170	1,0	3	17	6	39	150
17	61,8	37	100	949	190	2600	23,5	6	72	84	13	170	8,7	3	17	134	39	150
18	68,6	37	100	1999	190	2600	37,8	6	72	207	13	170	0,9	3	17	27	39	150
19	39,2	37	100	187	190	2600	33,9	6	72	79	13	170	2,4	3	17	11	39	150
20	32,3	37	100	169	190	2600	25,1	6	72	87	13	170	3,3	3	17	17	39	150
21	31,2	37	100	167	190	2600	17,5	6	72	94	13	170	13,7	3	17	73	39	150
22	67,5	37	100	764	190	2600	22,8	6	72	75	13	170	1,1	3	17	13	39	150
23	62,8	37	100	550	190	2600	5,8	6	72	18	13	170	0,4	3	17	4	39	150
24	55,0	37	100	219	190	2600	25,3	6	72	52	13	170	0	3	17	0	39	150
25	52,4	37	100	165	190	2600	13,3	6	72	25	13	170	5,4	3	17	17	39	150
26	47,1	37	100	149	190	2600	19,8	6	72	32	13	170	2,2	3	17	7	39	150
27	53,0	37	100	275	190	2600	26,3	6	72	66	13	170	5,4	3	17	28	39	150
28	50,7	41	81	393	200	1700	22,2	7	85	44	13	160	4,8	4	14	37	20	270
29	64,0	41	81	661	200	1700	32,0	7	85	116	13	160	7,2	4	14	74	20	270
30	49,8	41	81	241	200	1700	15,9	7	85	36	13	160	2,1	4	14	10	20	270
31	30,2	41	81	144	200	1700	26,0	7	85	82	13	160	3,2	4	14	15	20	270
32	30,0	31	81	85	150	1500	13,3	7	47	23	14	160	6,1	4	20	17	33	190
33	41,4	31	81	167	150	1500	11,3	7	47	27	14	160	0,2	4	20	1	33	190
34	39,5	31	81	148	150	1500	18,2	7	47	56	14	160	8,0	4	20	30	33	190
35	17,0	7,0	100,0	82	50	2400	23,2	5	56	70	24	190	2,2	4	17	11	25	180

No.	Tc CD3+CD8+						CD4:CD8			Naive Tc CD8+RA+CD197+CD27+					
	%	N. min	N. max	cc	N. min	N. max		N. min	N. max	%	N. min	N. max	cc	N. min	N. max
1	15	7	35	982	300	2700	0,86	0,6	5,6	18,3	44	100	179	140	2200
2	18	6	41	408	300	3400	2,63	1,0	4,0	38,7	47	100	158	290	1650
3	12	10	35	615	200	5400	3,71	1,2	3,5	39,1	31	100	241	150	3200
4	15	10	35	558	200	5400	1,67	1,2	3,5	62,9	31	100	351	150	3200
5	6	10	35	72	200	5400	4,25	1,2	3,5	63,2	31	100	45	150	3200
6	26	7	58	1235	400	4100	1,60	1,2	3,5	27,4	16	100	339	140	2460
7	17	7	58	326	400	4100	2,60	1,2	3,5	67,9	16	100	221	140	2460
8	22	7	58	467	400	4100	1,65	1,2	3,5	79,8	16	100	373	140	2460
9	38	7	58	954	400	4100	1,10	1,2	3,5	75,3	16	100	718	140	2460
10	13	7	58	605	400	4100	3,31	1,2	3,5	79,2	16	100	479	140	2460
11	14	7	40	411	200	2800	2,97	1,0	3,0	63,7	10	100	262	30	3100
12	27	7	40	1436	200	2800	1,41	1,0	3,0	55,3	10	100	795	30	3100
13	22	7	40	746	200	2800	1,76	1,0	3,0	31,0	10	100	231	30	3100
14	7	7	40	184	200	2800	2,48	1,0	3,0	75,3	10	100	138	30	3100
15	14	9	49	510	200	1800	2,86	1,0	2,1	53,6	19	100	274	53	1100
16	10	9	49	240	200	1800	2,65	1,0	2,1	58,9	19	100	141	53	1100
17	25	9	49	1185	200	1800	1,30	1,0	2,1	59,9	19	100	709	53	1100
18	27	9	49	1972	200	1800	1,48	1,0	2,1	68,2	19	100	1344	53	1100
19	10	9	49	213	200	1800	2,24	1,0	2,1	62,2	19	100	133	53	1100
20	5	9	49	125	200	1800	4,20	1,0	2,1	17,0	19	100	21	53	1100
21	15	9	49	676	200	1800	1,80	1,0	2,1	13,0	19	100	88	53	1100
22	41	9	49	1439	200	1800	0,79	1,0	2,1	11,6	19	100	167	53	1100
23	20	9	49	485	200	1800	1,77	1,0	2,1	75,6	19	100	374	53	1100
24	18	9	49	218	200	1800	1,83	1,0	2,1	51,0	19	100	111	53	1100
25	18	9	49	200	200	1800	1,58	1,0	2,1	43,1	19	100	86	53	1100
26	23	9	49	240	200	1800	1,32	1,0	2,1	22,8	19	100	55	53	1100
27	26	9	49	481	200	1800	1,08	1,0	2,1	46,5	19	100	224	53	1100
28	20	13	47	451	200	1700	1,72	1,2	2,7	70,9	16	100	320	42	1300
29	30	13	47	800	200	1700	1,63	1,2	2,7	39,7	16	100	318	42	1300
30	35	13	47	563	200	1700	0,86	1,2	2,7	46,1	16	100	259	42	1300
31	27	13	47	296	200	1700	1,61	1,2	2,7	19,5	16	100	58	42	1300
32	37	14	40	533	300	1300	0,53	1,2	2,7	8,4	20	95	45	78	640
33	18	14	40	127	300	1300	3,19	1,2	2,7	45,0	20	95	57	78	640
34	22	14	40	423	300	1300	0,89	1,2	2,7	27,7	20	95	117	78	640
35	41	12	40	691	200	1200	0,70	1,2	2,7	8,0	6	100	55	16	1000

No.	Terminally differentiated Tc CD8+RA+CD197- CD27-						Central memory Tc CD8+RA-CD197+CD27+						Effector memory Tc CD8+RA-CD197-CD27-					
	%	N. min	N. max	cc	N. min	N. max	%	N. min	N. max	cc	N. min	N. max	%	N. min	N. max	cc	N. min	N. max
1	5,7	3	42	56	15	480	1,5	2	35	15	13	380	8,7	1	23	85	9	190
2	3,5	3	40	14	13	820	3,5	0,1	24	14	1	190	3,4	0,5	70	14	2	400
3	0,4	5	78	9	17	2800	9,1	0,1	13	56	2	150	3,2	1	100	20	8	1400
4	1,5	5	78	8	17	2800	5,5	0,1	13	18	2	150	0,5	1	100	2	8	1400
5	4,6	5	78	3	17	2800	1,3	0,1	13	1	2	150	2,6	1	100	2	8	1400
6	10,9	4	92	135	29	1700	6,0	2	6	74	12	92	27,6	1	100	341	8	2100
7	1,7	4	92	6	29	1700	16,3	2	6	53	12	92	0,0	1	100	0	8	2100
8	1,5	4	92	7	29	1700	4,8	2	6	22	12	92	0,0	1	100	0	8	2100
9	0,2	4	92	2	29	1700	3,3	2	6	31	12	92	0,0	1	100	0	8	2100
10	3,4	4	92	21	29	1700	11,4	2	6	69	12	92	0,3	1	100	2	8	2100
11	1,6	8	71	7	55	460	8,5	1	8	35	5	110	0,7	2	100	3	16	630
12	3,2	8	71	46	55	460	10,5	1	8	151	5	110	1,1	2	100	15	16	630
13	10,3	8	71	77	55	460	6,5	1	8	48	5	110	7,3	2	100	54	16	630
14	0,0	8	71	0	55	460	16,1	1	8	30	5	110	4,3	2	100	8	16	630
15	17,6	6	83	90	25	530	0,7	1	9	4	4	64	3,2	10	55	16	24	590
16	2,5	6	83	6	25	530	6,8	1	9	16	4	64	0,0	10	55	0	24	590
17	0,7	6	83	9	25	530	5,5	1	9	65	4	64	0,5	10	55	6	24	590
18	1,9	6	83	38	25	530	6,2	1	9	122	4	64	0,3	10	55	5	24	590
19	1,2	6	83	2	25	530	5,2	1	9	11	4	64	0,6	10	55	1	24	590
20	8,1	6	83	10	25	530	16,6	1	9	17	4	64	2,2	10	55	2	24	590
21	26,7	6	83	180	25	530	51,7	1	9	349	4	64	6,2	10	55	42	24	590
22	33,0	6	83	475	25	530	0,9	1	9	13	4	64	34,8	10	55	500	24	590
23	2,2	6	83	11	25	530	3,4	1	9	17	4	64	0,4	10	55	2	24	590
24	0,0	6	83	0	25	530	5,1	1	9	11	4	64	3,4	10	55	7	24	590
25	15,0	6	83	30	25	530	3,0	1	9	6	4	64	3,2	10	55	6	24	590
26	5,4	6	83	13	25	530	3,5	1	9	8	4	64	7,4	10	55	18	24	590
27	12,8	6	83	62	25	530	4,9	1	9	24	4	64	2,9	10	55	14	24	590
28	4,4	15	41	20	57	340	6,3	1	6	28	6	43	2,7	5	100	12	45	410
29	2,1	15	41	17	57	340	4,9	1	6	39	6	43	1,7	5	100	13	45	410
30	10,4	15	41	59	57	340	4,7	1	6	27	6	43	2,2	5	100	12	45	410
31	18,9	15	41	56	57	340	11,8	1	6	35	6	43	11,5	5	100	34	45	410
32	25,8	9	65	138	35	420	0,7	0,4	18	4	2	86	24,4	4	100	130	16	810
33	0,2	9	65	1	35	420	13,0	0,4	18	17	2	86	2,8	4	100	4	16	810
34	21,0	9	65	89	35	420	2,7	0,4	18	11	2	86	6,1	4	100	26	16	810
35	21,0	7	53	145	25	280	3,1	1	20	21	5	120	32,1	14	98	222	40	640
